# Pleurisy

**Published:** 1894-05-12

**Authors:** 


### Medical Progress.

### PLEURISY.

Why does fluid effused into the pleura assume the peculiar S-shaped curve of Ellis?

This is fully and accurately explained by Le Fevre[1], who points out the erroneous statement of the majority of text books that the exudation gravitates to the most dependent portions of the pleural sac, and immediately exerts a pressure upon the diaphragm and thoracic wall, and forces the lower portion of the lung upward in direct proportion to the extent of the effusion. It is also taught in most of the books that the upper surface of the fluid, when the patient is standing or sitting erect, is in a horizontal plane, and, when the position is changed, there is a corresponding change in the level of the fluid; in other words, it acts as fluid in an open vessel, always seeking a hydrostatic level. This explanation, while very simple, entirely ignores the physiological laws of respiration, and takes no cognizance of the force that are involved.

Clinicians have repeatedly called attention to cases of pleurisy with effusion in which the line of flatness was not straight, and did not really change with the altered posture of the patient. These departures from the assumed typical line have been explained by many plausible theories.

Damorseau first discovered that the line of flatness was always a curved one when the lung on the affected side retained its normal elasticity. Ellis later emphasized the fact and accurately described the curve, so that to-day it is known as the curved line of Ellis, or the letter S line.

Garland (Pneumonodynamics), in a series of experiments on dogs by injections of cocoa butter into the pleural cavities, demonstrated, by the moulds thus obtained, that this curved line was constant when the lung was normal, and was formed by the action of the lung on the fluid, and not, as was generally supposed, by the fluid moulding the lung. He found that "the fluid collects first in the complemental spaces. Thence it extends in a narrow band upward and forward between the lower border of the lung and costal attachment of the diaphragm. At the same time it spreads out in a thin sheet over the diaphragm. The fluid continued to proceed in this manner until it began to bag down the diaphragm, to obliterate the intercostal depressions, and to rise behind the lung in the vertebral groove, and lastly, it appears between the lungs and the lateral chest wall."

The amount of distension of the normal lung during life is not as yet accurately determined. Foster, by fitting a manometer in the trachea of a cadaver and then opening the thorax found the pressure to be about seven millimetres of mercury. This is far below that of life. All portions of the lung are not equally distended, the distension being greatest in the lower segment, in the axillary region, less in the mammary, and least behind.

In pleurisy with effusion the fluid, if no other force acted upon it, would gravitate to the most dependent portion of the pleura and seek a hydrostatic level. But as the lung contracts with most force in the radii of its greatest distension, it is able to overcome or modify gravity and draw the fluid to that part, as was shown in the distribution of fluid in Garland's experiments, and the flatness on percussion will appear first in the axillary line. Portions of the lung will continue to draw the fluid until the distension is equal throughout; consequently the level of the fluid will rise higher at those points where the distension of the lung was the greatest.

Rokitansky says the normal lung can contract to an eighth of the size it has when distended in the thorax. Until the zero point of contraction is reached, the fluid exerts no compression on the lung. "It merely flees before the fluid." All excess of fluid beyond this point begins to exert a positive compression of the lung.

Thus far the reaction of the healthy lung only has been considered. If the lung is bound down by adhesions or its resilience is diminished by pathological conditions—as emphysema, tubercular infiltration, &c.—then the fluid would not assume as marked a curve line; it would earlier exert a positive pressure on the diaphragm and chest wall and a compressing effect on the lung. This line has a positive diagnostic value, as its curve depends entirely on the elasticity of the lung. Although foreign to the subject, says the author, the effect of the fluid on the lung sheds some light on the relation that the occurrence of an effusion into the pleural cavity has upon tubercular disease in the lower lobe of the lung on that side, often changing an acute process into a chronic one, or even arresting it.

*Treatment of Acute Pleurisy.*—Dr. Aufrecht[2] thinks the good results obtained in recent years in the treatment of pleurisy depend in great part upon the use of salicylic acid, of which he first wrote in 1883. His subsequent experience strengthens his earlier recommendation. But salicylic acid, not salicylate of sodium, must be used. The salicylate is weaker and produces marked secondary effects. When the pure acid is given in wafers patients must always be admonished to follow the dose with a copious draught of water, in order to prevent a disagreeable burning feeling in the stomach.

Aufrecht has adopted the following modification of his former method of giving the salicylic acid. If in the first eight days no diminution of the exudate occurs do not conclude that the salicylic acid has proved useless, but stop it for a day or two, and then begin again the dose of six grammes (90 grains) a day in divided doses of one gramme, and so continue the treatment for several days with interruptions of a day or two. Aufrecht adds that he has repeatedly found the same method of administration successful in obstinate fever.

Salicylic acid is not beneficial in all cases, particularly not in effusions secondary to tumors of the mediastinum and lung, carcinoma, or tuberculosis of the pleura, and in effusions of long standing. As soon as the salicylic acid proves ineffective, the fluid should be withdrawn by aspiration. Fever is not a contra-indication to the operation in tuberculous pleurisy. Dr. Osler[3] considers the indications for treatment are two-fold: (1) To limit and control the exudate and promote its absorption. In the early stage it is generally sufficient to allay pain, if severe, with opium and to reduce the fever, if high, by sponging, and to keep the bowels freely moved. He does not think the salicylates are deserving of the confidence claimed for them.

The effusion may be made to subside sometimes by a dry diet and brisk saline cathartics. Diuretin is sometimes useful. If, at the end of ten days the exudate persists to the extent of reaching the fourth rib in the erect posture, aspiration is called for, for it is important to relieve the lung early of the compression lest it becomes bound down by such firm adhesions that it cannot expand.

The second indication is to improve the general nutrition of the patient.

[1] New York Med. Journ. Feb. 24, 1894. [2] Ther. Monat. Sept. 1893; Mer. Gaz. Jan., 1894. [3] Trans. Mass. Med. Soc., 1893.

